# Identification and characterization of *RET* fusions in advanced colorectal cancer

**DOI:** 10.18632/oncotarget.4325

**Published:** 2015-05-30

**Authors:** Anne-France Le Rolle, Samuel J. Klempner, Christopher R. Garrett, Tara Seery, Eric M. Sanford, Sohail Balasubramanian, Jeffrey S. Ross, Philip J. Stephens, Vincent A. Miller, Siraj M. Ali, Vi K. Chiu

**Affiliations:** ^1^ Division of Hematology/Oncology, Department of Medicine, University of California Irvine, Irvine, CA, USA; ^2^ Chao Family Comprehensive Cancer Center, University of California Irvine, Orange, CA, USA; ^3^ The Division of Cancer Medicine, Department of Gastrointestinal Medical Oncology, MD Anderson Cancer Center, Houston, TX, USA; ^4^ Foundation Medicine Inc., Cambridge, MA, USA; ^5^ Albany Medical College, Albany, NY, USA

**Keywords:** RET fusion kinase, RET kinase inhibitor, comprehensive genomic profiling, colorectal cancer

## Abstract

There is an unmet clinical need for molecularly directed therapies available for metastatic colorectal cancer. Comprehensive genomic profiling has the potential to identify actionable genomic alterations in colorectal cancer. Through comprehensive genomic profiling we prospectively identified 6 *RET* fusion kinases, including two novel fusions of *CCDC6-RET* and *NCOA4-RET*, in metastatic colorectal cancer (CRC) patients. *RET* fusion kinases represent a novel class of oncogenic driver in CRC and occurred at a 0.2% frequency without concurrent driver mutations, including *KRAS*, *NRAS*, *BRAF*, *PIK3CA* or other fusion tyrosine kinases. Multiple RET kinase inhibitors were cytotoxic to *RET* fusion kinase positive cancer cells and not *RET* fusion kinase negative CRC cells. The presence of a *RET* fusion kinase may identify a subset of metastatic CRC patients with a high response rate to RET kinase inhibition. This is the first characterization of *RET* fusions in CRC patients and highlights the therapeutic significance of prospective comprehensive genomic profiling in advanced CRC.

## INTRODUCTION

Colorectal cancer (CRC) is the second most common cause of cancer-related death in the United States. Currently, metastatic CRC patients are treated mainly as an unselected cohort with angiogenesis inhibitors and cytotoxic chemotherapy. Only the absence of an oncogenic *KRAS* or *NRAS* mutation has been found to predict clinical benefit from treatment with anti-EGFR antibodies [1]. Although recent molecular characterization of CRC has not yet been translated into effective therapeutic strategies [2], comprehensive genomic profiling has emerged as a promising approach that enables the identification of genomic biomarkers that may inform the use of targeted therapy in clinical trials. This therapeutic genomic paradigm is best demonstrated in tumors that are driven by activated protein tyrosine kinases due to oncogenic mutations or rearranged chromosomal fusion [3]. Classic examples include gefitinib inhibition of *EGFR* mutant kinase in non-small cell lung cancer (NSCLC) and imatinib inhibition of *BCR-ABL* fusion kinase in chronic myeloid leukemia [4, 5].

Oncogenic *RET* point mutations and rearranged *RET* fusions induce hereditary and sporadic tumors [6]. *RET* fusion kinase occurs in nearly one-third of papillary thyroid cancer and ∼ 2% of lung adenocarcinoma, but is not yet identified in CRC [6]. *RET* fusion kinase juxtaposes the C-terminal *RET* kinase domain to an N-terminal coiled-coil or leucine zipper dimerization domain from multiple 5′ fusion partners to trigger ligand independent activation of downstream signaling pathways such as RAS-MAPK and PI3K-AKT [6, 7]. Here, we prospectively identified by comprehensive genomic profiling the presence of *RET* fusion kinase in CRC patients. Evidence of therapeutic response in CRC patient with a *CCDC6-RET* fusion treated with the RET kinase inhibitor regorafenib highlights the therapeutic importance of genomic profiling in colorectal cancer.

## RESULTS

### Characterization of *RET* fusions in CRC patients

**Figure 1:**
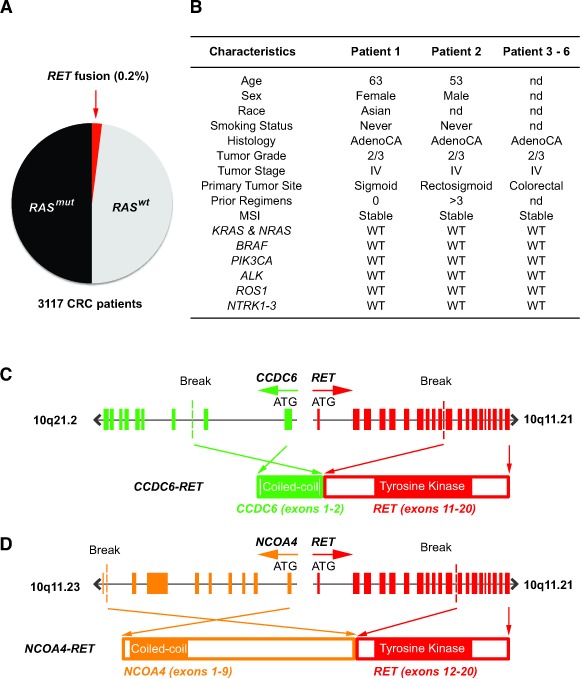
Characterization of *RET* fusions in CRC patients **A.** Frequency of *RET* fusions in unselected metastatic CRC patients as detected by NGS. **B.** Genetic and clinicopathologic characteristics of 6 patients harboring *RET* fusion kinase. nd = no data and WT = wild type. **C.** Fusion of *CCDC6* exon 11 (green) containing the coiled-coil domain to *RET* exon 11 (red) containing the tyrosine kinase domain to generate *CCDC6-RET* fusion kinase. **D.** Fusion of *NCOAT* exon 9 (orange) containing the coiled-coil domain to *RET* exon 12 (red) containing the tyrosine domain to generate *NCOAT-RET* fusion kinase.

To identify novel oncogenic drivers in colorectal cancer that may be targeted therapeutically, we performed prospective comprehensive genomic profiling using next generation sequencing (NGS) on metastatic colorectal tumors in the complete coding sequence of 236 cancer-related genes and the introns of 19 frequently rearranged cancer-related genes (Supplemental Table 1). Prior retrospective analyses with NGS of 40 CRC specimens detected a *C2orf44-ALK* fusion kinase but did not identify any *RET* fusion kinase [8]. As expected, we detected mutations in *KRAS*, *PIK3CA*, *BRAF*, *p53* and *APC*, which are known mutated oncogenes and tumor suppressors in CRC (data not shown). We prospectively profiled 3,117 metastatic colorectal tumors, and identified the presence of 6 *RET* fusion kinases to give a frequency of 0.2% (Figure 1A). The clinicopathologic characteristics of these six *RET* fusion-positive CRC patients revealed the absence of a concurrent driver mutation or other fusion tyrosine kinases (Figure 1B). We identified two novel *RET* fusion kinases involving 5′ fusion partners *Coiled Coil Domain Containing 6* (*CCDC6*) and *Nuclear Receptor Coactivator 4* (*NCOA4*) in two patients with metastatic CRC. Patient 1 had a *CCDC6-RET* fusion kinase with amino-terminal *CCDC6* exon 1-2 and carboxyl-terminal *RET* exon 11-19 (Figure 1C). The *CCDC6-RET* fusion kinase in patient 1 occurred at a novel breakpoint at *CCDC6* intron 2 and *RET* intron 10 from a chromosome 10 inversion event, which differs from the *CCDC6-RET* fusion kinase breakpoints in thyroid cancer and lung cancer that fused *CCDC6* exon 1 to *RET* exon 12-20 [9]. Patient 2 had an *NCOA4-RET* fusion kinase with amino-terminal *NCOA4* exons 1-9 and carboxyl-terminal *RET* exons 12-19, with breakpoints at *NCOA4* intron 9 and *RET* intron 11 from a chromosome 10 tandem duplication event (Figure 1D). The *NCOA4*α isoform observed in patient 2 was nearly full length in comparison to the truncated *NCOA4*β isoform fused to *RET* exon 12 in papillary thyroid carcinoma and NSCLC adenocarcinoma [10, 11].

### Cytotoxic effect of RET kinase inhibitors in *RET* fusion-positive cancer cells

**Figure 2:**
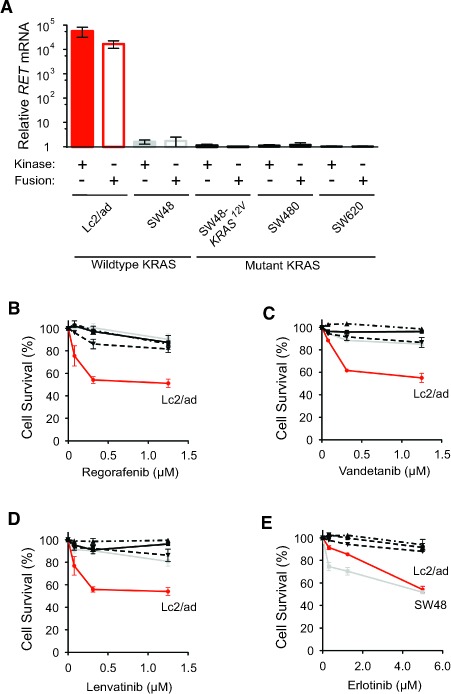
Inhibition of *RET* fusion-positive cancer cells viability by RET kinase inhibitors **A.** Relative *RET* mRNA levels in Lc2/ad, SW48, SW48,-KRAS^12V^, SW480, and SW620 cells as measured by quantitative RT-PCR analysis and normalized to SW620 cells using primers that recognized the *RET* kinase domain (Kinase: +) or flanked the *CCDC6-RET* fusion site (Fusion: +). **B.**-**E.** Lc2/ad, SW48, SW48,-KRAS^12V^, SW480, and SW620 cells were treated with indicated concentrations of regorafenib **B.** vandetanib **C.** lenvatinib **D.** and erlotinib **E.** for 72 hours and cell survival was determined relative to 0.1% DMSO-treated controls (mean ± STD; *n* = 3).

Because regorafenib inhibits the proliferation of thyroid TT cells driven by oncogenic *RET ^C634W^* point mutation [12], we next tested the ability of regorafenib to inhibit *RET* fusion-positive cancer cells. Using two different primer pairs that either bound within the *RET* kinase domain or flanked the *CCDC6-RET* fusion site, we confirmed by quantitative PCR that Lc2/ad cancer cells, but not several KRAS wildtype and mutant CRC cells, harbored the *CCDC6-RET* fusion kinase (Figure 2A) [13]. Treatment with nanomolar concentration of regorafenib was cytotoxic to Lc2/ad cells, but the *RET* fusion-negative CRC cells remained resistant even at micomolar concentration (Figure 2B). Vandetanib and lenvatinib, which are FDA-approved RET kinase inhibitors with clinical efficacy against *RET* fusion-positive thyroid cancer [14, 15], followed the same pattern and specifically inhibited only Lc2/ad cells viability (Figure 2C and 2D). In contrast, both KRAS wildtype Lc2/ad and SW48 cells had increased sensitivity to erlotinib treatment, and KRAS mutant CRC cells were resistant to this EGFR kinase inhibitor as predicted (Figure 2E) [16]. RET kinase inhibitors specifically suppressed only *RET* fusion-positive cancer cells viability and did not show non-specific suppression of *RET* fusion-negative CRC cells viability with either KRAS wildtype or mutant status.

### Clinical response of *RET* fusion-positive CRC patient to regorafinib

**Figure 3:**
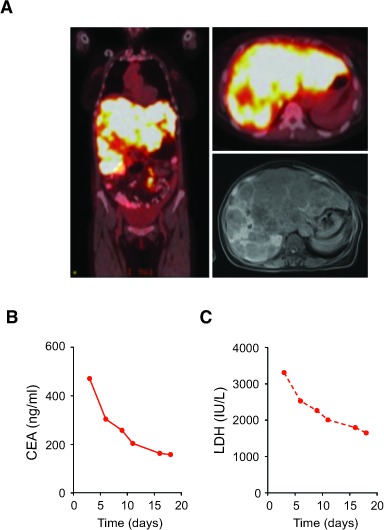
Clinical response of *RET* fusion-positive CRC patient to regorafinib **A.** Scans of Patient 1 harboring *CCDC6-RET* fusion kinase with diffuse liver metastases evident on PET/CT scan on coronal (left) and transverse (upper right) sections and MRI scan on transverse section (lower right). **B.** Serum CEA of patient 1 treated with regorafenib 40-80 mg daily. **C.** Serum LDH of Patient 1 treated with regorafenib 40-80 mg daily.

The absence of a concurrent driver mutation in *RET* fusion-positive CRC patients suggest that they may respond to a RET kinase inhibitor. Patient 1 was a 63-year-old woman who was diagnosed with stage IV sigmoid colon adenocarcinoma, and the PET/CT and MRI scans showed widespread metastases most notable for an enlarged liver that was nearly replaced by tumor (Figure 3A). Patient 1 declined chemotherapy-based treatment and wished to minimize therapy related toxicity. She was treated with low-dose regorafenib 80 mg daily for 3 days and continued at 40 mg daily. Clinically, patient 1 had resolution of her early satiety and abdominal discomfort within 1 week of regorafenib initiation. She had a rapid CEA response from 471 to 158 and LDH response from 3310 to 1651 after 18 days of treatment (Figures 3B-3C). No further follow-up was available as the patient succumbed to urosepsis shortly thereafter. Her clinical and CEA tumor marker responses suggested regorafenib has single agent activity in *RET* fusion positive CRC.

### Colorectal cancer classification based on genomic biomarkers

**Figure 4:**
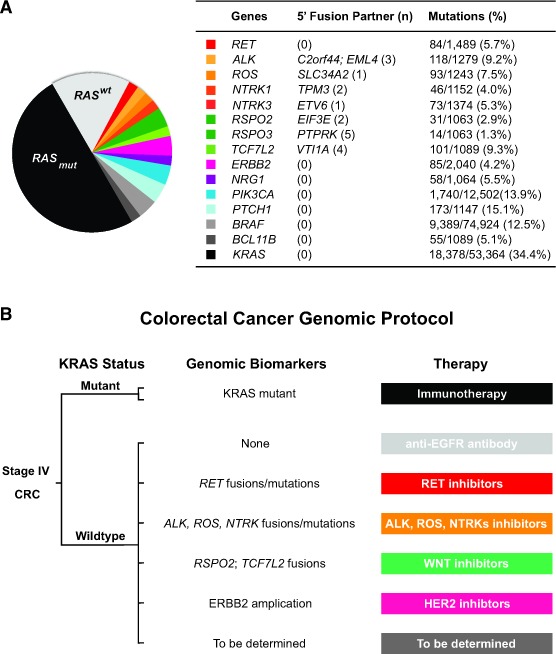
Colorectal cancer classification based on genomic biomarkers **A.** Schematic classification of colorectal cancer based on genomic biomarkers with accompanying table showing fusion partner numbers (n) and gene point mutations available from the COSMIC database. **B.** Schematic of a Colorectal Cancer Genomic Protocol for stage IV CRC patients with assignment of therapy based on specific genomic biomarkers.

Next, we analyzed the Catalogue of Somatic Mutations in Cancer (COSMIC) database for cancer-related gene fusions and point mutations in CRC patients. In addition to the six *RET* fusion kinases we have identified, there were other fusion kinases involving *ALK*, *NTRK* and *ROS* and WNT pathway fusions involving *RSPO* and *TCF7L2* (Figure 4A) [8, 17-21]. Based on the COSMIC database, genetic alterations by rearranged fusions were less common than missense or nonsense point mutations in those genes. *RET* point mutations occurred in 5.7% of CRC patients, and 30 of the 84 *RET* point mutations were localized within the RET kinase domain (Figure 4A). Notably, 8 *RET* mutations occurred specifically at E768, R844, S904, R912 and M918 (data not shown), which corresponded to hereditary *RET* mutated sites in familiar medullary thyroid carcinoma and multiple endocrine neoplasia type 2B [6, 22].

## DISCUSSION

Using comprehensive genomic profiling we have identified six *RET* fusion kinases in CRC and provide early clinicopathologic characteristics of this patient subset. The *RET* fusion kinase appears to be a mutually exclusive oncogenic driver that does not overlap with other known driver mutations such as *KRAS*, *BRAF*, *EGFR* and *PIK3CA* and oncogenic fusion kinases involving *ALK*, *ROS1* and *NTRK*. Since we observed a low 0.2% frequency in unselected CRC patients, this mutual exclusivity may facilitate prospective screening for *RET* fusion kinase in CRC patients who are pan-negative for other known driver mutations. Further, the breakpoint locations observed on two of our patients appear to be novel. Our clinicopathologic observation that *RET* fusion kinase occurs in younger never smoker patients without other known driver alterations is consistent with observations in fusion kinase driven NSCLC and will require further validation in a larger cohort of CRC patients [11, 23].

Our data suggest that CRC patients who harbor *RET* fusion kinase without a concurrent driver mutation may respond to regorafenib, a potent RET kinase inhibitor with an IC_50_ of ∼ 1.5n M [12]. The *in-vitro* studies suggest low dose regorafenib potently suppresses *RET* fusion-positive cancer cells but has minimal inhibitory activity in *RET* fusion-negative CRC cells. Indeed, only 1% of unselected metastatic CRC patients treated with regorafenib had an objective or partial response in the CORRECT trial, which demonstrated a 1.4 month overall survival benefit compared to placebo [24]. We cannot preclude the possibility that the clinical activity of regorafenib in the CORRECT trial may be due to its inhibition of other kinase targets such as PDGFR or VEGFR, and *RET* fusion status analysis of the 1% regorafenib responders is needed. Since these *RET* fusion-positive CRC patients received a high median dose of regorafenib 147 mg daily, additional potential off-target effects likely contributed to the 54% incidence rate of severe or life-threatening adverse events [24]. Notably, *RET* fusion-positive cancer cells, but not *RET* fusion-negative cancer cells, had similar exquisite sensitivity to regorafenib, vandetanib and lenvatinib, which share RET and VEGFR kinases as the only common molecular targets. In light of the poor single agent activity of angiogenesis inhibitor in CRC patients, our findings suggest that the presence of *RET* fusion kinase identifies a subset of CRC patients with exceptional response to RET kinase inhibition. Further work and larger series are required to confirm and expand upon our findings.

Treatment of metastatic CRC patients with regorafenib at the maximum tolerated dose as used in the CORRECT trial has significant toxicity, and low-dose regorafenib may be of sufficient potency to inhibit tumors driven by *RET* fusion kinase [24]. To minimize therapy related toxicity and improve anti-RET kinase response, it is worthwhile to evaluate the therapeutic index of currently available anti-RET tyrosine kinase inhibitors in the subset of CRC patients who express *RET* fusion kinase. Although there may be a tissue-specific or contextual contribution to response, our findings are similar to reported responses to RET-directed therapies in thyroid cancer and NSCLC. Currently, several tyrosine kinase inhibitors with anti-RET kinase activity such as vandetanib, lenvatinib, ponatinib, and carbozantinib are at various stages of clinical development for medullary thyroid carcinoma, in which *RET* is the critical oncogenic driver, and lung adenocarcinoma. Vandetanib, cabozantinib, and lenvatinib are FDA-approved as treatment in advanced thyroid cancer, since they significantly prolonged progression-free survival when compared to placebo [14, 15, 25]. In *RET*-fusion positive NSCLC patients who had progressed on prior chemotherapy, treatment with the RET kinase inhibitors, vandetanib or carbozantinib, results with partial responses in three patients [26, 27]. We now extend the clinical activity of RET fusion kinase inhibition to CRC patients.

Our analysis of the COSMIC database summarizes the frequency of oncogenic fusion kinases and point mutations in CRC patients. However, the COSMIC database most likely underestimates the frequency of gene fusions and point mutations in CRC patients since the entire coding sequence of each gene was not determined. Using a comprehensive genomic profiling approach, we prospectively identified 6 *RET* fusions in CRC and an overall *RET* fusion frequency of 0.2%. The identification of CRC patients with actionable *RET* fusion kinase provides further evidence of the impact that NGS has on clinical decision-making, and we anticipate the rapid adoption of prospective genomic profiling as a part of standard practice. The availability of NGS facilitates a therapeutic genomic paradigm to classify CRC based on actionable genomic biomarkers such as *RET*, *ALK*, *NTRK*, *ROS* and *ERBB2*, which may facilitate the clinical trial development of a Colorectal Cancer Genomic Protocol (Figure 4B).

## MATERIALS AND METHODS

### Next generation sequencing

Next-generation sequencing (NGS) assay covering 3,769 exons of 236 cancer-related genes and 47 introns of 19 genes frequently rearranged in cancer (Supplemental Table 1) was performed by Foundation Medicine, Inc., a CLIA-certified and CAP-accredited laboratory, based on a modified published protocol.[8] Briefly, formalin-fixed, paraffin-embedded (FFPE) specimen quality of volume >1mm^3^, nucleated cellularity >80% or >30,000 cells, and >20% tumor nuclei was ensured by macro-dissection as needed and confirmed by a pathologist. DNA was extracted using the Promega Maxwell 16 Tissue LEV DNA kit and quantified using an Invitrogen Picogreen fluorescence assay. Library Construction was performed with 50-200 ng of DNA sheared by sonication (Covaris E210) to ∼100-400 bp before end-repair, dA addition, ligation of indexed Illumina sequencing adaptors and PCR amplification for 10 cycles using Kapa HiFi. Solution phase hybridization was performed with a custom baitset of 120-bp biotinylated DNA oligonucleotides (Integrated DNA Technology), and 49 × 49 paired-end sequencing was performed using the Illumina HiSeq 2000 and Illumina HiSeq 2500 platforms. Sequence alignment, PCR duplicate read removal, and local alignment optimization was performed using BWA aligner 0.5.9, Picard 1.47 (http://picard.sourceforge.net/), Samtools 0.1.12a, and GATK 1.0.4705, and variant calling was performed using custom tools. Base substitutions were called using a Bayesian methodology, short indels were called using local assembly, copy number alterations were called through comparison to process-matched normal controls, and rearrangements were called using chimeric read pairs clustered by genomic position. Somatic variants were annotated using COSMIC and germline variants were removed using dbSNP.

### Patients, cell lines and reagents

Informed consents for diagnostic testing and therapy were obtained from patients involved in this study. SW48, SW480 and SW620 cells were obtained from American Type Culture Collection (ATCC; Manassas, VA). Lc2/ad cells were obtained from Sigma-Aldrich (St. Louis, MO). We transduced SW48 cells with pCCL-KRAS^12V^ lentivirus to obtain SW48-KRAS^12V^ cells. All cells were thawed from frozen stocks expanded from original cells obtained from ATCC and cultured for less than 3 months in Dulbecco's Modified Eagle Medium supplemented with 10% fetal bovine serum. Regorafenib, vandetanib and lenvatinib were obtained from Selleck pharmaceutical (Houston, TX).

### Quantitative PCR

Purified mRNA from human tumor cells using a Hi-Pure RNA isolation kit (Roche Life Science) were reverse transcribed with SuperScript Reverse Transcriptase II (Invitrogen) to generate cDNA template. Quantitative PCR was performed on the Roche Lightcycler 480 II with the addition of Taq polymerase, SYBR green (Roche Life Science) and primer pairs (Fisher Scientific). *RET-* forward primer (5′-GGCTTGTCCCGAGATGTTTA-3′) and *RET* reverse primer (5′-TCTTTTGGTGTCCTGCTGTG-3′) recognized the *RET* tyrosine kinase domain, and *CCDC6-197* forward primer (5′-TGCAGCAAGAGAACAAGGTG-3′) and *RET-2318* reverse primer (5′-CAGGCCCCATACAATTTGAT-3′) flanked the *CCDC6-RET* fusion site.

### Cell toxicity assay

A total of 8,000 cells/well were plated in 100μl of culture media in 96-well plates and treated the next day with 100μl of 0.1% DMSO media control or drugs at the indicated concentrations. After 72 hours, AlamarBlue (Life Technologies) was added, and cellular fluorescence was quantified per manufacturer instruction with a BioTEK Synergy 2 microplate reader.

## SUPPLEMENTARY MATERIAL TABLE


