# Supplementary material for: Identification and characterization of RET fusions in advanced colorectal cancer
Source: Oncotarget. 2015 May 30;6(30):28929–37. doi: 10.18632/oncotarget.4325 (PMC4745701; doi:10.18632/oncotarget.4325)
Supplement: Supplementary file 1 [file oncotarget-06-28929-s001.pdf]

Identification and characterization of RET fusions in advanced colon cancer

Supplementary Material

SUPPLEMENTAL TABLE 1

FOUNDATION ONE Gene List

ABL1	BTK	CTNNB1	FGF23	IL7R	MLH1	PDGFRA	SMO
AKT1	CARD11	DAXX	FGF3	INHBA	MLL	PDGFRB	SOCS1
AKT2	CBFB	DDR2	FGF4	IRF4	MLL2	PDK1	SOX10
AKT3	CBL	DNMT3A	FGF6	IRS2	MPL	PIK3CA	SOX2
ALK	CCND1	DOT1L	FGFR1	JAK1	MRE11A	PIK3CG	SPEN
APC	CCND2	EGFR	FGFR2	JAK2	MSH2	PIK3R1	SPOP
AR	CCND3	EMSY (C11orf30)	FGFR3	JAK3	MSH6	PIK3R2	SRC
ARAF	CCNE1	EP300	FGFR4	JUN	MTOR	PPP2R1A	STAG2
ARFRP1	CD79A	EPHA3	FLT1	KAT6A (MYST3)	MUTYH	PRDM1	STAT4
ARID1A	CD79B	EPHA5	FLT3	KDM5A	MYC	PRKAR1A	STK11
ARID2	CDC73	EPHB1	FLT4	KDM5C	MYCL1	PRKDC	SUFU
ASXL1	CDH1	ERBB2	FOXL2	KDM6A	MYCN	PTCH1	TET2
ATM	CDK12	ERBB3	GATA1	KDR	MYD88	PTEN	TGFBR 2
ATR	CDK4	ERBB4	GATA2	KEAP1	NF1	PTPN11	TNFAIP 3
ATRX	CDK6	ERG	GATA3	KIT	NF2	RAD50	TNFRS F14
AURKA	CDK8	ESR1	GID4 (C17orf39)	KLHL6	NFE2L2	RAD51	TOP1
AURKB	CDKN1B	EZH2	GNA11	KRAS	NFKBIA	RAF1	TP53
AXL	CDKN2A	FAM123B (WTX)	GNA13	LRP1B	NKX2-1	RARA	TSC1
BAP1	CDKN2B	FAM46C	GNAQ	MAP2K1	NOTCH1	RB1	TSC2
BARD1	CDKN2C	FANCA	GNAS	MAP2K2	NOTCH2	RET	TSHR
BCL2	CEBPA	FANCC	GPR124	MAP2K4	NPM1	RICTOR	VHL
BCL2L2	CHEK1	FANCD2	GRIN2A	MAP3K1	NRAS	RNF43	WISP3
BCL6	CHEK2	FANCE	GSK3B	MCL1	NTRK1	RPTOR	WT1
BCOR	CIC	FANCF	HGF	MDM2	NTRK2	RUNX1	XPO1
BCORL1	CREBBP	FANCG	HRAS	MDM4	NTRK3	SETD2	ZNF217
BLM	CRKL	FANCL	IDH1	MED12	NUP93	SF3B1	ZNF703
BRAF	CRLF2	FBXW7	IDH2	MEF2B	PAK3	SMAD2	
BRCA1	CSF1R	FGF10	IGF1R	MEN1	PALB2	SMAD4	
BRCA2	CTCF	FGF14	IKBKE	MET	PAX5	SMARCA4	
BRIP1	CTNNA1	FGF19	IKZF1	MITF	PBRM1	SMARCB1	

FOUNDATION ONE Selected Rearrangements

ALK	BRAF	ETV4	EWSR1	NTRK1	RARA	TMPRSS2	
BCR	EGFR	ETV5	MLL	PDGFRA	RET		
BCL2	ETV1	ETV6	MYC	RAF1	ROS1		
